# Discovery of an Ebolavirus-Like Filovirus in Europe

**DOI:** 10.1371/journal.ppat.1002304

**Published:** 2011-10-20

**Authors:** Ana Negredo, Gustavo Palacios, Sonia Vázquez-Morón, Félix González, Hernán Dopazo, Francisca Molero, Javier Juste, Juan Quetglas, Nazir Savji, Maria de la Cruz Martínez, Jesus Enrique Herrera, Manuel Pizarro, Stephen K. Hutchison, Juan E. Echevarría, W. Ian Lipkin, Antonio Tenorio

**Affiliations:** 1 National Center of Microbiology, (ISCIII), Madrid, Spain; 2 Center for Infection and Immunity, and WHO Collaborating Centre for Diagnostics, Surveillance and Immunotherapeutics for Emerging Infectious and Zoonotic Diseases, Mailman School of Public Health of Columbia University, New York, New York, United States of America; 3 Grupo Asturiano para el Estudio y Conservacion de los Murciélagos, Posada de Llanera, Principado de Asturias, Spain; 4 Evolutionary Genomics Laboratory, Centro de Investigación Príncipe Felipe, Valencia, Spain; 5 Evolutionary Biology Unit, Estación Biológica Doñana (CSIC), Sevilla, Spain; 6 Service of Pathology, Veterinary Teaching Hospital, Veterinary School, Complutense University, Madrid, Spain; 7 Roche Life Sciences, Branford, Connecticut, United States of America; Mount Sinai School of Medicine, United States of America

## Abstract

Filoviruses, amongst the most lethal of primate pathogens, have only been reported as natural infections in sub-Saharan Africa and the Philippines. Infections of bats with the ebolaviruses and marburgviruses do not appear to be associated with disease. Here we report identification in dead insectivorous bats of a genetically distinct filovirus, provisionally named Lloviu virus, after the site of detection, Cueva del Lloviu, in Spain.

## Author Summary

A novel filovirus, provisionally named Lloviu virus (LLOV), was detected during the investigation of *Miniopterus schreibersii* die-offs in Cueva del Lloviu in southern Europe. LLOV is genetically distinct from other marburgviruses and ebolaviruses and is the first filovirus detected in Europe that was not imported from an endemic area in Africa. Filoviruses, amongst the most lethal of primate pathogens, have only been reported as natural infections in sub-Saharan Africa and the Philippines. Infections of bats with the ebolaviruses and marburgviruses do not appear to be associated with disease. Here we report identification of genetically distinct filovirus in dead insectivorous bats in caves in Spain.

## Introduction

Filoviruses cause lethal hemorrhagic fever in humans and nonhuman primates. The family *Filoviridae* includes two genera: *Marburgvirus*, comprising various strains of the Lake Victoria marburgvirus (MARV); and *Ebolavirus* (EBOVs), comprising four species including Sudan ebolavirus (SEBOV), Zaire ebolavirus (ZEBOV), Ivory Coast ebolavirus (CIEBOV), and Reston ebolavirus (REBOV); and a tentative species Bundibugyo ebolavirus (BEBOV) [1]. MARV was discovered in 1967 in Marburg, Germany during an outbreak in laboratory staff exposed to tissues from monkeys imported from Uganda. ZEBOV was discovered in 1976 in Yambuku, Zaire during a 312-person outbreak associated with 90% mortality. With the exception of REBOV, that appears to be pathogenic in nonhuman primates but not in humans and is endemic in the Philippines, all known filoviruses are pathogenic in primates including humans and are endemic in Africa [2]. Bats are implicated as reservoirs and vectors for transmission of filoviruses in Africa [3]. ZEBOV sequences have been found in fruit bats (*Hypsignathus monstrosus, Epomops franqueti and Myonycteris torquata*) [4], [5]. MARV sequences have been found in fruit (*Rousettus aegyptiacus*) and insectivorous (*Rhinolophus eloquens and Miniopterus inflatus*) bats [6], [7]. Bats naturally or experimentally infected with ZEBOV or MARV are healthy and shed virus in their feces for up to 3 weeks [4], [5], [7].

In 2002, colonies of Schreiber's bats (*Miniopterus schreibersii*), sustained massive die-offs in caves in France, Spain and Portugal [8]. *M. schreibersii*, family *Vespertilionidae*, comprises at least four geographically discrete lineages distributed in Oceania, southern Europe, southern Africa, and southeast Asia [9]. Here we report the discovery of a novel ebolavirus-like filovirus in bats from Europe.

## Results

**Figure 1:**
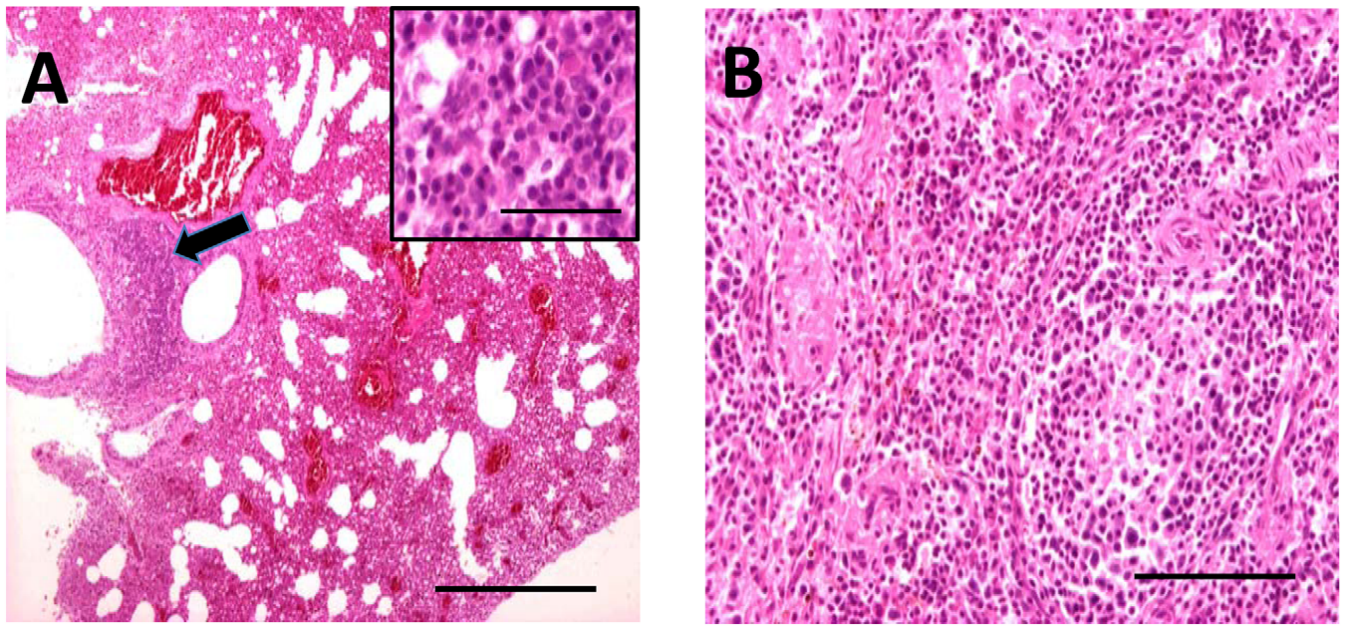
Hematoxylin and eosin stained sections through lung (A) and spleen (B) of infected *M. schreibersii*. (A) Thickened interalveolar septae (arrowhead) (bar  = 500 µm) and infiltrates comprising lymphocytes and macrophages (higher magnification inset) (bar  = 50 µm). (B) Depletion of the lymphocytes and lymphoid follicles (bar  = 200 µm).

Bat carcasses from Cueva del Lloviu, Asturias, Spain (5° 32′ 8.1′ ´ N and 43° 30′ 5.6′ ´ W) were collected for anatomical, microbiological and toxicological analyses. Although no gross pathology was apparent, microscopy of internal organs revealed interstitial lung infiltrates comprised of lymphocytes and macrophages, and depletion of lymphocytes and lymphoid follicles in spleen (**Figure 1** **)**. These findings were consistent with viral pneumonia; hence, nucleic acid from lung and spleen were analyzed by consensus polymerase chain reaction (PCR) for the presence of a broad range of viral agents including lyssa-, paramyxo-, henipa-, corona-, herpes- and filoviruses. Filovirus sequences were detected in extracts from lung, liver, rectal swabs or spleen of 5 animals. Pairwise distance analysis of the 186 nucleotide product showed highest similarity with ZEBOV (73.7%). A sensitive real time PCR assay established to quantitate viral burden confirmed the presence of filoviral sequences in the original 5 animals and from an additional 15 with similar pathology collected from the same cave (**Table 1**). A liver sample with the highest viral load by PCR (4.0×10^6^ genome copies/gr) was selected for high-throughput sequencing yielding 225,758 reads that represented 12.1 kilobases of viral sequence. Gaps between fragments and genomic termini were completed by specific PCR and rapid amplification of cDNA ends (RACE) to obtain a nearly complete genome.

**Table 1:** Quantification of LLOV by TaqMan Real Time PCR.

Sample	Location	Bat species	Throat Swab (copies/µL)	Rectal Swab (copies/µL)	Spleen (copies/gr)	Brain (copies/gr)	Lung (copies/gr)	Liver (copies/gr)
67	Lloviu	*M. schreibersii*	ND	ND	ND	ND	2.3×10^6^	ND
68	Lloviu	*M. schreibersii*	ND	ND	ND	ND	4.8×10^5^	ND
69	Lloviu	*M. schreibersii*	ND	ND	ND	ND	1.9×10^6^	ND
70	Lloviu	*M. schreibersii*	ND	ND	ND	ND	8.8×10^4^	ND
71	Lloviu	*M. schreibersii*	ND	ND	ND	ND	1.2×10^5^	ND
72	Lloviu	*M. schreibersii*	ND	ND	ND	ND	1.1×10^4^	ND
73	Lloviu	*M. schreibersii*	ND	ND	ND	ND	3.4×10^5^	ND
74	Lloviu	*M. schreibersii*	ND	ND	ND	ND	3.3×10^5^	ND
75	Lloviu	*M. schreibersii*	ND	ND	ND	ND	3.8×10^5^	ND
76	Lloviu	*M. schreibersii*	ND	ND	ND	ND	8.4×10^5^	ND
78	Lloviu	*M. schreibersii*	ND	Negative	ND	ND	3.2×10^4^	ND
79	Lloviu	*M. schreibersii*	ND	Negative	ND	ND	3.6×10^4^	ND
80	Lloviu	*M. schreibersii*	ND	Negative	ND	ND	2.0×10^5^	ND
81	Lloviu	*M. schreibersii*	ND	Negative	ND	ND	4.2×10^4^	ND
82	Lloviu	*M. schreibersii*	ND	Positive	3.1×10^4^	ND	5.8×10^4^	4.0×10^4^
83	Lloviu	*M. schreibersii*	ND	Positive	9.4×10^4^	ND	6.2×10^4^	ND
84	Lloviu	*M. schreibersii*	ND	Negative	2.3×10^5^	ND	4.5×10^5^	1.1×10^5^
85	Lloviu	*M. schreibersii*	ND	Positive	2.0×10^3^	ND	1.1×10^6^	ND
86	Lloviu	*M. schreibersii*	ND	Positive	9.9×10^5^	ND	5.7×10^5^	4.0×10^6^
87	LLoviu	*M. schreibersii*	ND	ND	ND	ND	6.9×10^4^	ND
129	Cantabria	*M. schreibersii*	1.9×10^2^	5.2×10^1^	ND	2.2×10^2^	1.8×10^4^	4.1×10^3^
130	Cantabria	*M. schreibersii*	8.4×10^3^	5.7×10^2^	ND	1.3×10^4^	4.4×10^5^	1.4×10^4^
131	Cantabria	*M. schreibersii*	2.9×10^2^	1.6×10^4^	ND	2.6×10^2^	3.5×10^4^	3.0×10^3^
132	Cantabria	*M. schreibersii*	1.3×10^2^	2.0×10^3^	ND	3.0×10^3^	ND	ND
133	Cantabria	*M. schreibersii*	5.2×10^2^	6.7×10^2^	ND	2.1×10^2^	7.0×10^3^	2.3×10^2^
134	Cantabria	*M. myotis*	Negative	ND	ND	Negative	Negative	Negative
135	Cantabria	*M. myotis*	Negative	Negative	ND	ND	Negative	Negative
136	Cantabria	*M. myotis*	Negative	Negative	ND	Negative	Negative	Negative
137	Cantabria	*M. myotis*	Negative	Negative	ND	Negative	Negative	Negative
138	Cantabria	*M. myotis*	Negative	ND	ND	Negative	ND	ND
139	Cantabria	*M. myotis*	Negative	Negative	ND	Negative	Negative	Negative
140	Cantabria	*M. myotis*	Negative	Negative	ND	Negative	Negative	Negative
141	Cantabria	*M. myotis*	Negative	Negative	ND	Negative	Negative	Negative
142	Cantabria	*M. myotis*	Negative	Negative	ND	Negative	Negative	Negative

ND; not done/not available.

Reports of bat die-offs in additional caves prompted analysis of a second set of samples from caves in Cantabria, Spain, wherein many dead *M. schreibersii* were observed. Throat and rectal swabs, brain, lung and liver were collected from five dead *M. schreibersii,* and nine dead *Myotis myotis*. Whereas filovirus sequences were detected by real time PCR in all *M. schreibersii* samples, no filovirus sequences were found in the *M. myotis* (**Table 1**). Real time PCR analysis of throat swabs and stool samples from 1,295 healthy bats representing 29 different bat species (including 45 healthy *M. schreibersii* from Lloviu cave collected after the die-offs) collected in several geographic locations in Spain revealed no evidence of filovirus infection (**Figure S1**).

Sequencing of regions of the L and NP genes of the original Lloviu Cave bat samples resulted in nearly identical sequences to the prototype sequence; a similar lack of variation was observed within each lineage of MARV in fruit bat reservoirs in the Kitaka cave, Uganda, although in that instance two clearly differentiated lineages were observed [7].

**Figure 2:**
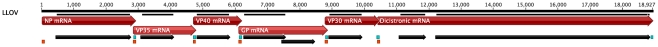
Genomic organization of LLOV. The black bars indicate the ORFs while the red arrows corresponding to their predicted mRNA transcripts. Start (turquoise) and termination (orange) signals for each transcript are displayed.

Consistent with the genomic organization characteristic of filoviruses, Lloviu virus (LLOV), named for the cave in which it first was found, has a 19 kb negative sense, single stranded RNA genome that contains seven open reading frames (ORF) (GenBank Accession number JF828358). However, LLOV differs from other filoviruses in transcriptional features. Analysis of conserved transcriptional initiation and termination sites suggests that the seven LLOV ORFs are encoded by six mRNA transcripts, one of which is dicistronic and contains both the VP24 and the L ORF (**Figure 2**). Additionally, although the LLOV termination signal is identical to ebolaviruses, the LLOV initiation signal is unique (3′-CUUCUU(A/G)UAAUU-5′). Several attempts by RACE to obtain complete genomic sequence were unsuccessful. By analogy to other filoviruses we assume that up to 700 nt may be missing at the 5′ terminus of the genome. This assumption is based on the observation that all known negative-strand RNA viruses have complementary termini and that length of noncoding sequences at the termini of filoviruses do not exceed 700 nt.

LLOV sequence was analyzed for similarities to EBOVs and MARV. In EBOVs a C-terminal basic amino acid motif in VP35 mediates type I interferon antagonism by binding to double-stranded RNA and inhibiting RIG-I signaling. This domain is conserved in LLOV VP35 (**Figure S2**). In non-segmented, negative strand RNA viruses, matrix proteins are not only key structural components of the virions, but also play important roles in the maturation and cellular egress steps of the viral life cycles. Short amino-acid sequences, termed late-budding motifs or L domains, are crucial for these events. The matrix protein in EBOVs, encoded by VP40, has overlapping P(T/S)AP and PPXY late-budding motifs at the N-terminus [10], [11] and YXXL late-budding motifs in the C-terminus. MARV VP40s contains only PPXY motifs. LLOV contains only a PPXY motif in the N-terminal domain of the VP40; hence, in this aspect, it is more similar to MARV than to EBOVs. The filovirus GP2 has an immunosuppressive motif [12], [13] (**Figure S3**); this motif is highly conserved in LLOV. EBOV VP24 interacts with the KPNα proteins that mediate PY-STAT1 nuclear accumulation [14]. Two domains of VP24 are required for inhibition of IFN-β-induced gene expression and PY-STAT1 nuclear accumulation (region 36–45 and 142–146) [15]. LLOV VP24 ORF has significant homology to EBOV VP24s; however, interaction domains are not well conserved (**Figure S4**, shaded areas).

**Figure 3:**
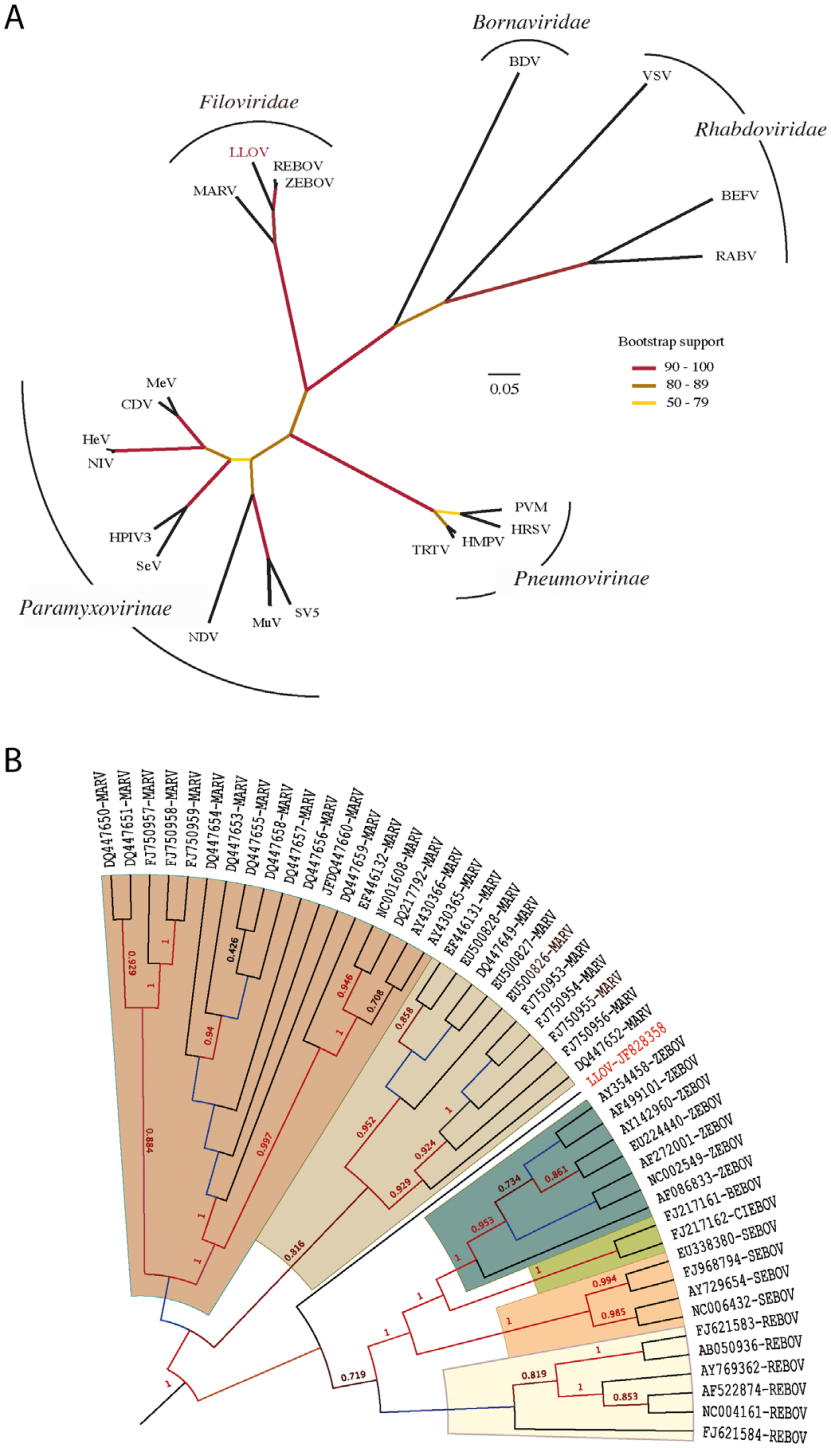
Phylogenetic analysis of LLOV. (**A**) **Analysis of the conserved domain of the RNA-dependent RNA polymerase of** ***Mononegavirales*** **.** Branch lengths in the unrooted tree are nonsynonymous distances (dN) taken from the subset of the second codon position of the conserved domain III of the polymerase protein (DS1). Bootstrap results (displayed in colors) were computed using 1,000 pseudoreplicates of the original dataset (DS1); (**B**) **Analysis of the complete genome of** ***Filoviridae*** **.** Rooted topology summarizes the historical relationships of 48 complete genome viruses of *Filoviridae*. Values on branches represent clade probabilities (SH). Values lower than 0.5 are not shown. Branch lengths were constrained to show ultrametric distances. Unconstrained distances and the full set of outgroup species are shown in **Figure S5**.

Phylogenetic analysis of conserved domain III of the RNA-dependent RNA polymerase demonstrates that LLOV belongs to the *Filoviridae* and may represent a complex of viruses related to all EBOVs (**Figure 3A**). Phylogenetic analysis of complete genome sequences (∼21,800 nucleotides) confirmed that LLOV is a distinct genetic lineage that originates after MARV **(** **Figure 3B** **)**. Bayesian and ML phylogenetic analyses using 7 outgroup species supported these conclusions (**Figure S5**).

MARV and EBOV are proposed to have diverged 7,100–7,900 years ago [16]. The inclusion of LLOV and use of Bayesian methods suggests a most recent common ancestor for all filoviruses ∼155,500 years ago (95% HPD of 87,375–249,630 years) and divergence of EBOVs and LLOV approximately 68,400 years ago (95% HPD of 38,857–109,460 years).

MARV and EBOV genomes differ by more than 50% at the nucleotide level. MARV genomes also differ from EBOV genomes in that they have only one, rather than several instances of gene overlap [17]. Whereas the MARV gene four (GP) encodes only one protein, the spike glycoprotein GP_1,2_, the EBOV gene four encodes proteins (sGP, Δ-peptide, GP_1,2_, ssGP) via transcriptional polymerase stuttering that results in frame shifts and, in the case of sGP/Δ-peptide, proteolytic processing [18], [19], [20], [21]. MARV spike proteins are highly N- and O-glycosylated but lack sialic acids, whereas EBOV spike proteins may contain sialic acids. Based on these differences, MARV and EBOV are assigned to two different genera. LLOV differs at the nucleotide level from MARVs by 57.3–57.7% and from EBOVs by 51.8–52.6% (**Figure S5**). The LLOV contains four instances of gene overlap and is predicted to express six transcripts rather than the seven observed in EBOV and MARV. Like EBOV, LLOV gene four (GP) possesses three overlapping ORFs coding for sGP/Δ-peptide, GP_1,2_ and ssGP analogs while maintaining the proteolytic site that would generate the Δ-peptide. The product is predicted to be highly N- and O-glycosylated. Given these features, LLOV represents the prototype of a new genus, tentatively designated *Cuevavirus* [17].

## Discussion

Although the dynamics of epidemic filoviral diseases among humans, great apes, and other primates have been described in detail [22], [23], [24], the natural reservoirs, modes of transmission to hominids and pongids (gorillas, and chimpanzees), and temporal dynamics remain obscure. Life forms of diverse taxa have been suggested as potential reservoirs, including bats, rodents, arthropods, and plants [5], [25]. Several lines of evidence support a role for bats including virus replication at high levels in experimentally inoculated insectivorous bats [5]; asymptomatic infection of fruit and insectivorous bats with EBOV in central Africa [4]; asymptomatic infection of fruit and insectivorous bats with MARV [6], [26]; and a history consistent with human exposure to a fruit bat reservoir during a ZEBOV outbreak in the Democratic Republic of Congo (DRC) in 2007 [27].

The discovery of LLOV in *M. schreibersii* is consistent with filovirus tropism for bats. However, unlike MARV and EBOV, where asymptomatic circulation is posited to be consistent with evolution to avirulence in this long-term host-parasite relationships, several observations suggest that in the case of LLOV, filovirus infection may be pathogenic. LLOV was found in the affected bat population (*M. schreibersii*) but not in other healthy *M. schreibersii* or in bats of other species that cohabited the same caves (*M. myotis*). Furthermore, lung and spleen, tissues with evidence of immune cell infiltrates consistent with viral infection, contained LLOV RNA sequences (**Table 1**).

The sudden outbreak of bat die-offs in Spain that precipitated this study destroyed several bat colonies in less than 10 days [8]. As recently highlighted by the example of white nose syndrome, a lethal fungal skin infection that is associated with recent declines in North American bat populations [28], bats play critical ecological roles in insect control, plant pollination, and seed dissemination. Although we have not demonstrated a causal relationship between LLOV and mortality in *M. schreibersii*, the discovery of a novel filovirus in western Europe, and the wide geographical distribution of the associated insectivorous bat is a significant concern. While the virus was detected in the north of Spain, simultaneous bat die-offs also have been observed in Portugal and France [8]. Filoviruses had been posited to show a geographically related phylogeographic structure [29]. Viruses and subtypes from particular geographic area cluster together phylogenetically, suggesting a stable host-parasite relationship wherein viruses are maintained in permanent local-regional pools. Whereas EBOV is associated with humid afrotropics, MARV is focused in drier areas in eastern and south-central Africa [29]. In that analysis, CIEBOV and ZEBOV coincided ecologically, while MARV, a more distantly related filovirus, did not. *M. schreibersii* distribution does not overlap with the predicted or observed areas of ZEBOV or MARV activity. Thus, LLOV appears not to share the known ecological or geographical niches of other recognized filoviruses.

Recently, the discovery of integrated filovirus elements has led to the proposal that filoviruses have co-evolved with mammals over millions of years [30], [31]. Phylogenetic analyses of LLOV indicate a common ancestor of all filoviruses at least 150,000 years ago. The discovery of a novel filovirus in a distinct geographical niche suggests that the diversity and distribution of filoviruses should be studied further.

## Materials and Methods

### Ethics statement

The study was made under projects SAF2006-12784-C02-02 and SAF2009-09172 approved by the General Research Program of the Spanish Government. Processed samples came from death bat carcasses collected from the floor of the caves. Sample collection was performed under special permit 14.03.443F (c.p. 1994-01680) from Principado de Asturias and regulation 32/1990 and 68/1995 from the “Dirección de Recursos Naturales y Protección Ambiental de la Consejería de Medio Ambiente del Principado de Asturias” and Royal Decree 439/1990. Sample collection in Cantabria was approved by the “Dirección General de Montes y Conservación de la Naturaleza” at the “Consejería de Agricultura y Ganadería y Pesca” under register E/07505.

### Samples

Thirty-four bat carcasses (25 *M. schreibersii*; 9 *M. myotis*) were collected during the bat die-offs occurring in 2002. Throat and rectal swabs, spleen, brain, lung and liver were stored when available. Then, during the period 2004–2008, rectal and throat swabs were obtained from 1295 healthy bats representing 29 different species (including *M. schreibersii* from distinct geographic locations in Spain)(**Figure S1**).

### Pathology

Six *M. schreibersii* bats were sent to the Service of Pathology of the Veterinary Teaching Hospital of the Veterinary School of the Complutense University of Madrid. During the course of necropsies no macroscopic lesions were observed, and samples for microbiology were obtained. Likewise, samples from the most significant organs and tissues were fixed in 10% buffered formalin for histology, embedded in paraffin and stained with hematoxylin and eosin.

### PCR

Amplification was carried out in a PCT-200 Peltier thermal cycler (MJ Research, Watertown, MA, USA) utilizing thin-walled reaction tubes (REAL, Durviz, Valencia, Spain). cDNA was obtained with SuperScript III RNase H Reverse transcriptase kit (Invitrogen SA, Spain/Portugal, Barcelona, Spain). A degenerate consensus PCR method for filovirus developed at the Instituto de Salud Carlos III, Madrid, was used for detection of the filovirus RNA-dependent RNA polymerase. Specific primers and protocols can be obtained from the authors on request. DNA bands of the correct size were purified using QIAquick Gel Extraction Kit (Qiagen) and sequenced using standard protocols (Applied Biosystems). After detection of filoviral sequences, primer-walking techniques utilizing degenerate primers on the L and NP gene were also used to obtain additional sequences of the genome (up to 2.5 kb).

### Genomic characterization of LLOV by high-throughput sequencing

Total RNA was extracted from the selected liver sample by using the Trizol procedure (Invitrogen, Carlsbad, CA, USA). Total RNA extracts were treated with DNase I (DNA-free, Ambion, Austin, TX, USA) and cDNA generated by using the Superscript II system (Invitrogen) for reverse transcription primed by random octamers that were linked to an arbitrary defined 17-mer primer sequence as previously described in detail [32]. The resulting cDNA was treated with RNase H and then randomly amplified by the polymerase chain reaction (PCR); applying a 9∶1 mixture of a primer corresponding to the defined 17-mer sequence and the random octamer-linked 17-mer primer, respectively. Products >70 base pairs (bp) were selected by column purification (MinElute, Qiagen, Hilden, Germany) and ligated to specific linkers for sequencing on the 454 Genome Sequencer FLX (454 Life Sciences, Branford, CT, USA) without fragmentation of the cDNA. Removal of primer sequences, redundancy filtering, and sequence assembly were performed with software programs accessible through the analysis applications at the CII Portal website (http://www.cii.columbia.edu). When traditional BLASTN, BLASTX and FASTX analysis failed to identify the origin of the sequence read, we applied FASD [33], a novel method based on the statistical distribution of oligonucleotide frequencies. The probability of a given segment belonging to a class of viruses is computed from their distribution of oligonucleotide frequencies in comparison with the calculated for other segments. A statistic measure was developed to assess the significance of the relation between segments. The p-value estimates the likelihood that an oligonucleotide distribution is derived from a different segment. Thus, highly related distributions present a high p-value.

After detection of several pieces of the genome of LLOV, specific PCR amplifications were performed to fill the gaps. Conventional PCRs were performed with HotStar polymerase (Qiagen) on PTC-200 thermocyclers (Bio-Rad, Hercules, CA, USA): an enzyme activation step of 5 min at 95°C was followed by 45 cycles of denaturation at 95°C for 1 min, annealing at 55°C for 1 min, and extension at 72°C for 1 to 3 min depending on the expected amplicon size. Amplification products were run on 1% agarose gels, purified (MinElute, Qiagen), and directly sequenced in both directions with ABI PRISM Big Dye Terminator 1.1 Cycle Sequencing kits on ABI PRISM 3700 DNA Analyzers (Perkin-Elmer Applied Biosystems, Foster City, CA).

### Data set and alignments

Three alternative data sets were analyzed in the study. The Mononegavirales data set 1 (hereafter DS1) collected 609 cDNA-aligned characters from the conserved domain III of the L gene along 21 species of the order. The filovirus data set 2 (DS2) collected the complete genome (21,794 aligned nucleotides) of 48 viruses of the family. The mononegaviral data set 3 (DS3) collected 19 genomes of filoviruses, and 7 genomes of pneumoviruses and paramyxoviruses used as outgroups to root the tree. In this case a total of 8,547 aligned characters from the L gene were used. For DS1 and DS2 alignments the corresponding polymerase protein sequence data were used as references. All DS were aligned using Muscle v3.7 (http://www.drive5.com/muscle/).

### Phylogenetic analyses

To override distance saturation in the mononegaviruses DS1, the conservative Ka distance was estimated for a subset of 303 second codon positions. Neighbor-Joining (NJ) tree, and 1,000 bootstrap pseudo-replicates were used to evaluate the tree support. Distances estimation, bootstrap and tree reconstruction were performed with SeaView 4.0 [34].

Filoviruses in particular (DS2) and mononegaviruses in general *(*DS3) were analyzed using maximum-likelihood (ML), and Bayesian methods of phylogenetic reconstruction. In both cases GTR+Γ fitted the parameters of the evolutionary model with the best AIC support. MrBayes v3.1.2 [35] was run using 1,000,000 generations for the filoviruses DS2, and 500,000 generations for the mononegaviruses DS3. In both cases sampling was done every 1,000 generations. To summarize topologies and parameters we retained the last 300 and 200 samples on each data set (which were 600 and 400 samples for DS2 and DS3 considering the two parallel runs of MrBayes). Markov chain Monte Carlo (MCMC) convergence was assessed by checking the average standard deviation of split frequencies (below 0.01) during more than 10,000 generations. Maximum-likelihood (ML) phylogenies were computed in PhyML v3.0 (http://www.atgc-montpellier.fr/phyml/). Shimodaira-Hasegawa (SH) test, and 500 pseudo-replicates of bootstrap analyses were computed to measure the statistical support of ML trees in the two data sets. Bayesian and ML topologies agreed upon the definition of the main clades of the phylogeny. Tree representations were prepared with FigTree V1.3.1.

### TMRCA

Using DS2, we also inferred a Maximum Clade Credibility (MCC) tree using the Bayesian Markov Chain Monte Carlo (MCMC) method available in the Beast package [36], thereby incorporating information on virus sampling time. This analysis utilized a strict molecular clock and a GTR+Γ model of nucleotide substitution for each codon position, although very similar results were obtained using other models. The analysis used a Bayesian skyline model as a coalescent prior. All chains were run until convergence for all parameters with 10% removed as burn-in.

### Real time PCR

Quantitative assays were established based upon virus specific sequences obtained from the high throughput sequencing for LLOV. A TaqMan real time PCR assay on the L gene was developed (primers available on request).

### Accession number

Genbank accession number JF828358 is available online through NCBI (http://www.ncbi.nlm.nih.gov/).

## Supporting Information

Figure S1 **Geographic locations of sites of specimen capture in Spain.** Bat capture locations are marked in red; LLOV positive sites are marked in blue.
(TIF)

Figure S2 **Conserved domains in the VP35 of filoviruses.** The NP and L binding domains, homo-oligomerization signals, and carboxy IID domain are all shown. The purple arrow represents the type-I interferon antagonist motif.
(TIF)

Figure S3 **Conserved domains in the GP1 of filoviruses.** Signal sequences (dark pink arrow), cysteines (yellow rectangles), glycosylation sites (purple triangles), and the immunosuppressive domain (purple arrow) are all shown.
(TIF)

Figure S4 **Conserved domains in the VP24 of filoviruses.** The two domains that are required for inhibition of IFN-β-induced gene expression and PY-STAT1 nuclear accumulation are shaded in red.
(TIF)

Figure S5 **Mononegavirales and the rooted position of LLOV.** LLOV locates as a sister group of the monophyletic cluster of the *Ebolavirus* after Bayesian and ML analysis of DS3. Values on internal branches denote bipartition support according with BY/BS/SH: Bayesian posterior probabilities, ML support using bootstrap, and ML-SH probabilities. Note the contrasted branch lengths displayed in the two main clusters of *Filoviridae*.
(TIF)
