# Supplementary figures and images for: Discovery of an Ebolavirus-Like Filovirus in Europe
Source: PLoS Pathog. 2011 Oct 20;7(10):e1002304. doi: 10.1371/journal.ppat.1002304 (PMC3197594; doi:10.1371/journal.ppat.1002304)

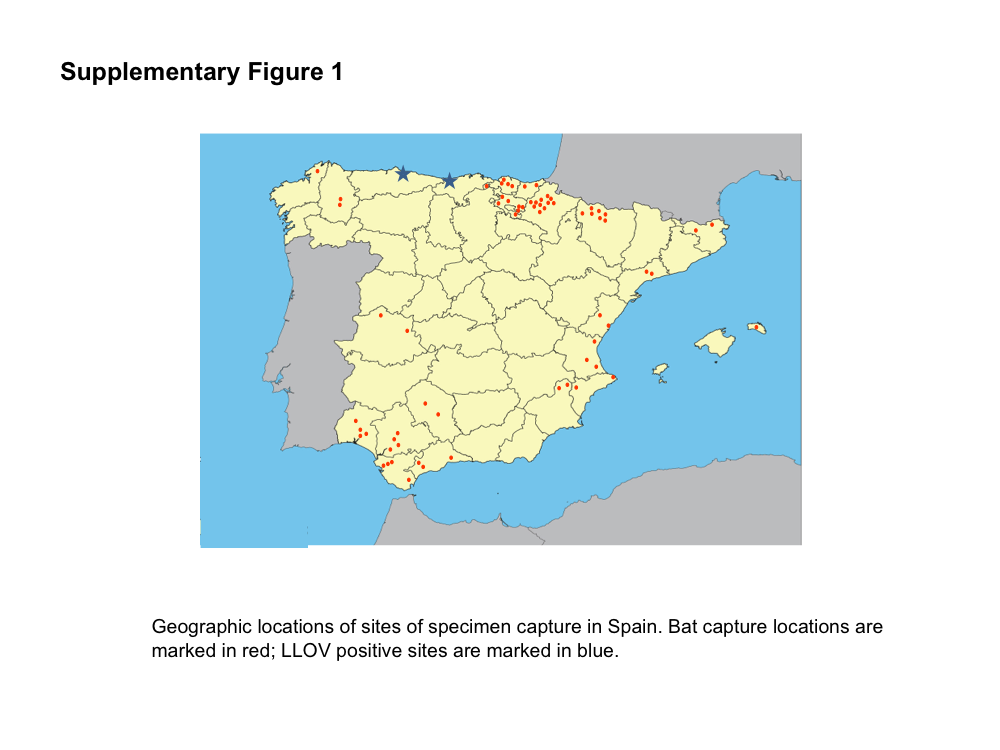

Supplement: Figure S1 — Geographic locations of sites of specimen capture in Spain. Bat capture locations are marked in red; LLOV positive sites are marked in blue. (TIF) [file ppat.1002304.s001.tif]

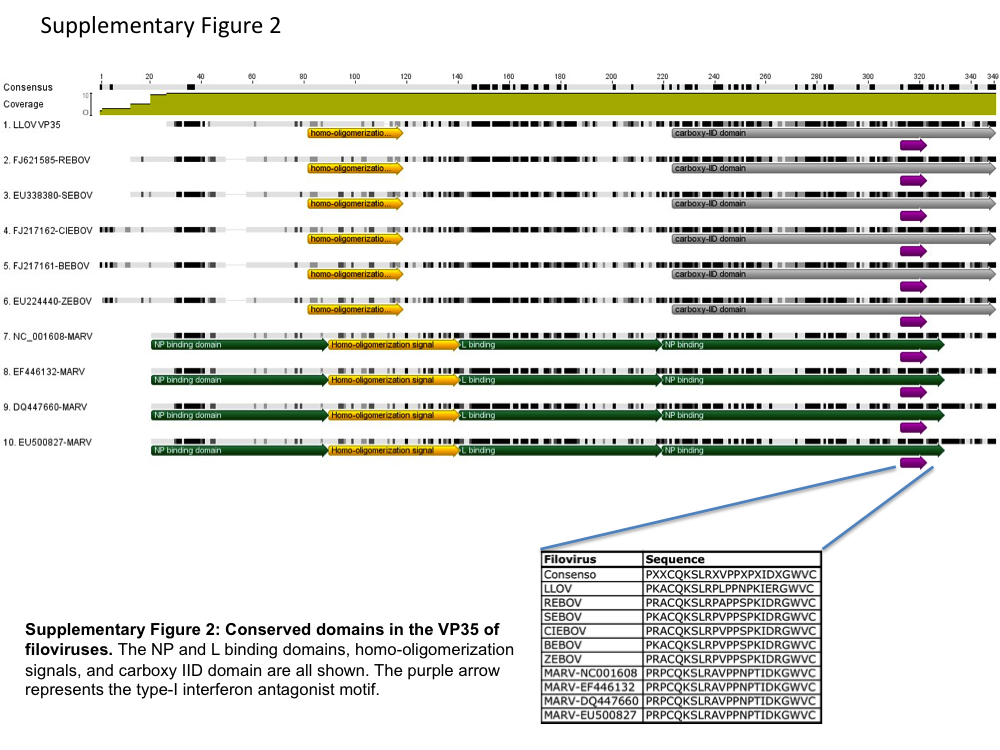

Supplement: Figure S2 — Conserved domains in the VP35 of filoviruses. The NP and L binding domains, homo-oligomerization signals, and carboxy IID domain are all shown. The purple arrow represents the type-I interferon antagonist motif. (TIF) [file ppat.1002304.s002.tif]

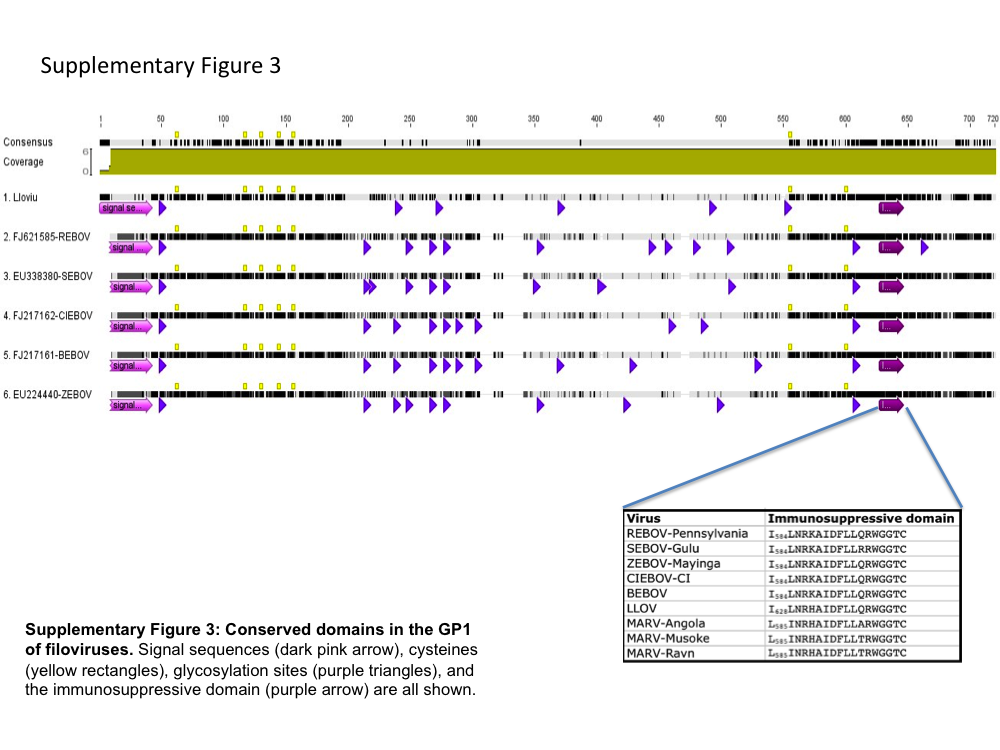

Supplement: Figure S3 — Conserved domains in the GP1 of filoviruses. Signal sequences (dark pink arrow), cysteines (yellow rectangles), glycosylation sites (purple triangles), and the immunosuppressive domain (purple arrow) are all shown. (TIF) [file ppat.1002304.s003.tif]

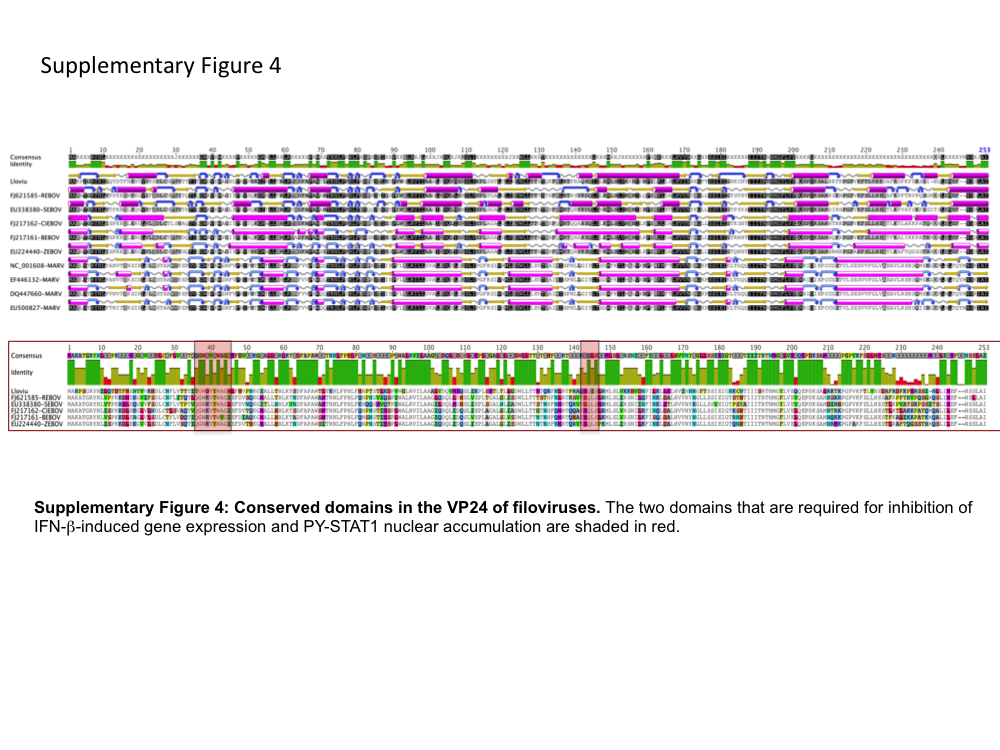

Supplement: Figure S4 — Conserved domains in the VP24 of filoviruses. The two domains that are required for inhibition of IFN-β-induced gene expression and PY-STAT1 nuclear accumulation are shaded in red. (TIF) [file ppat.1002304.s004.tif]

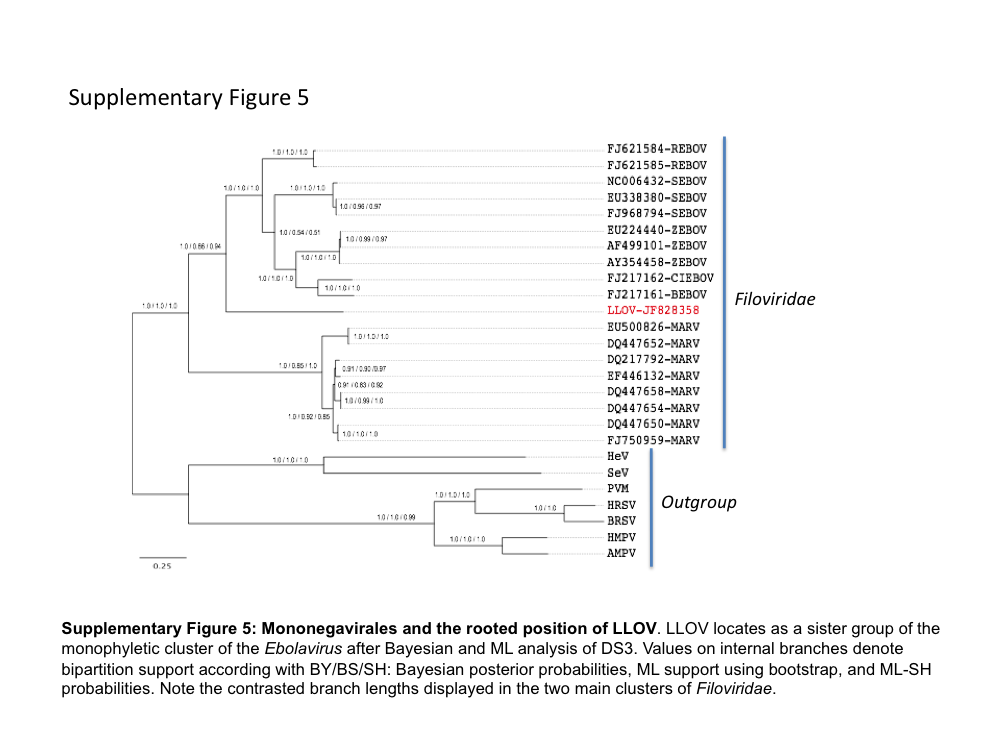

Supplement: Figure S5 — Mononegavirales and the rooted position of LLOV. LLOV locates as a sister group of the monophyletic cluster of the Ebolavirus after Bayesian and ML analysis of DS3. Values on internal branches denote bipartition support according with BY/BS/SH: Bayesian posterior probabilities, ML support using bootstrap, and ML-SH probabilities. Note the contrasted branch lengths displayed in the two main clusters of Filoviridae. (TIF) [file ppat.1002304.s005.tif]
